# The barriers dentists face to communicate cancer diagnosis: self-assessment based on SPIKES protocol

**DOI:** 10.4317/medoral.25650

**Published:** 2022-12-24

**Authors:** Beatriz Nascimento Figueiredo Lebre Martins, César Augusto Migliorati, Ana Carolina Prado Ribeiro, Manoela Domingues Martins, Thais Bianca Brandão, Marcio Ajudarte Lopes, Carolina Guimarães Bonfim Alves, Alan Roger Santos-Silva

**Affiliations:** 1 DDS, MSc. University of Campinas (UNICAMP), Oral Diagnosis Department, Piracicaba Dental School, Piracicaba, Brazil; 2 DDS, MSc, PhD. College of Dentistry, University of Florida, Florida, USA; 3 DDS, MSc, PhD. University of Campinas (UNICAMP), Oral Diagnosis Department, Piracicaba Dental School, Piracicaba, Brazil; São Paulo State Cancer Institute (ICESP-FMUSP), Dental Oncology Service, São Paulo, Brazil; 4 DDS, MSc, PhD. University of Campinas (UNICAMP), Oral Diagnosis Department, Piracicaba Dental School, Piracicaba, Brazil; Federal University of Rio Grande do Sul, Department of Oral Pathology, School of Dentistry, Rio Grande do Sul, Brazil; 5 DDS, MSc, PhD. São Paulo State Cancer Institute (ICESP-FMUSP), Dental Oncology Service, São Paulo, Brazil; 6 DDS, MSc, PhD. University of Campinas (UNICAMP), Oral Diagnosis Department, Piracicaba Dental School, Piracicaba, Brazil; 7 DDS, MSc. University of Campinas (UNICAMP), Oral Diagnosis Department, Piracicaba Dental School, Piracicaba, Brazil; São Paulo State Cancer Institute (ICESP-FMUSP), Dental Oncology Service, São Paulo, Brazil

## Abstract

**Background:**

This study aimed to characterize the barriers faced by Brazilian dentists to deliver bad news (DBN) about oral and oropharyngeal cancer diagnoses to patients by using a questionnaire based on the guidelines of the SPIKES protocol.

**Material and Methods:**

This was an observational cross-sectional study. The questionnaire contained 27 questions based on the SPIKES protocol, which were answered in the SurveyMonkey platform.

**Results:**

A total of 186/249 dentists answered the questionnaire. The main specialties reported were 36.02% oral medicine, 21.5% oral pathology, and 9.13% oral and maxillofacial surgery. A total of 44.6% expressed concern about the patient’s emotional reactions, and 46.24% of respondents had never participated in any specific training to communicate bad news.

**Conclusions:**

The lack of training and low confidence in dealing with patients’ emotional reactions dentists were considered the greatest barriers to DBNs. Moreover, most dentists who participated in the survey believe that a protocol to guide the communication of bad news would be useful for clinical practice. For those protocols to be used by dentists, training is critical for these protocols to be incorporated by professionals.

** Key words:**Deliver bad news, dentists, communication in health, oral cancer.

## Introduction

The literature shows that a general dentist working in Brazil for approximately 35 years sees an average of 5 patients with oral cancer manifestations. The National Cancer Institute, in Brazil, estimates approximately 15,190 new cases of oral and oropharyngeal cancer between 2020 and 2022, ranking fifth among males’ most prevalent cancers in the country. Additionally, most head and neck cancers (HNC) have a late diagnosis (1). In this scenario, how the diagnosis communication is made can influence the patients’ reaction when receiving a cancer diagnosis (2-4). Studies have shown that the adequate provision of information is proportional to the reduction in patients’ levels of anxiety and depression (5,6). In addition, developing good communication skills with patients can also reflect positively on the mental health of professionals (7).

Challenging conversations in oncology are, in many ways, similar to complex intervention procedures, since they require careful planning and execution, using well-developed strategies to facilitate proper communication (3,8). To this end, the literature reports a series of strategies to support best practices in delivering bad news (DBN), such as the ABCDE by Rabow and McPhee (9), and the SPIKES protocol (10), on which our study was based.

Over the past decade, several communication skills training courses for health professionals have emerged, especially for those who deal with cancer patients. This kind of training seems to better prepare professionals to deal with all aspects of delivering bad news to patients in a more suiTable way (10). Awojobi *et al*. (2016) reported that the training had a positive impact, reducing the barriers between professionals and patients and raising their self-confidence levels when discussing the diagnosis of oral cancer with patients (11).

Analysis of the main difficulties faced by dentists when they must deliver bad news can guide the development of training programs and communication strategies for undergraduate students and professionals. To our knowledge, no study has assessed strategies for DBN in Brazilian patients diagnosed with oral and oropharyngeal cancer. Our study used a questionnaire based on the SPIKES protocol to evaluate the barriers faced by Brazilian dental professionals when communicating cancer diagnoses to their patients (10).

## Material and Methods

Our study was performed in Brazil, with a collaboration between Piracicaba Dental School, University of Campinas (UNICAMP), and the Brazilian Society of Oral Medicine and Oral Pathology (SOBEP). It was approved by the local Ethics Committee for Human Studies, according to the recommendations of the National Health Council - Ministry of Health of Brazil for research involving human subjects (protocol number 32475120.6.0000.5418).

- Sample and Data Collection

This was a cross-sectional, quantitative study with the application of a questionnaire developed based on the guidelines of the SPIKES protocol to measure dentists’ level of confidence when communicating cancer diagnoses to patients. The sample consisted of dentists in Brazil who had to DBN of oral and/or oropharyngeal cancer diagnosis at least once during their clinical practice. The sample size was estimated using the SurveyMonkey calculator. The number of dentists registered in the Brazilian Federal Board of Dentistry (343,195 until October 2020), a 95% sampling confidence level (certainty) and a 10% margin of error (a small margin of error is close to the exact answer at a given confidence level) were considered. A total of 97 participants were needed.

One initial round of emails was sent to 78 dentists and current and former members of the Brazilian Society of Stomatology (SOBEP). This association concentrates on professionals of different dental specialties who communicate oncological diagnosis. The questionnaire invitations were sent via their institutional email and contained a link directing the participant to a consent form and an online questionnaire with 27 items based on the SPIKES protocol (Supplement 1). The questionnaire was answered in the “SurveyMonkey platform (SurveyMonkey Inc. San Mateo, CA, USA)”. At the end of the questionnaire, each participant had the option of forwarding the survey link to other professionals inviting them to participate in the survey, independent from their association with SOBEP, using the “snowball sampling” technique.

- Statistical analysis

Descriptive statistical analyses were performed based on the frequency, mean, standard deviation, and proportion of the evaluated sample. The Kruskal-Wallis test with Dwass-Steel-Critchlow-Fligner (DSCF) pairwise comparisons posttest, Chi-Square test, and Spearman correlation were performed to evaluate the influence of sociodemographic variables, place of work, time since graduation, specialty, and frequency on the time of needing to DBN to patients and on the confidence levels in communicating the cancer diagnosis. All analyses were performed with Jamovi software version 1.6, and statistical significance was set at *p*<0.05 with 95% confidential intervals.

## Results

The survey response rate was 74.6%. Table 1 shows the demographic profile of dentists. A total of 186 dentists completed the questionnaire; 75 (40.32%) were male, and 111 (59.67%) were female. The specialties reported were 67 (36.02%) oral medicine, 40 (21.5%) oral pathology, 17 (9.13%) oral and maxillofacial surgery, 32 (25.89%) other specialties, and 30 (16.12%) general dental practitioners (nonspecialists).

**Table 1:**
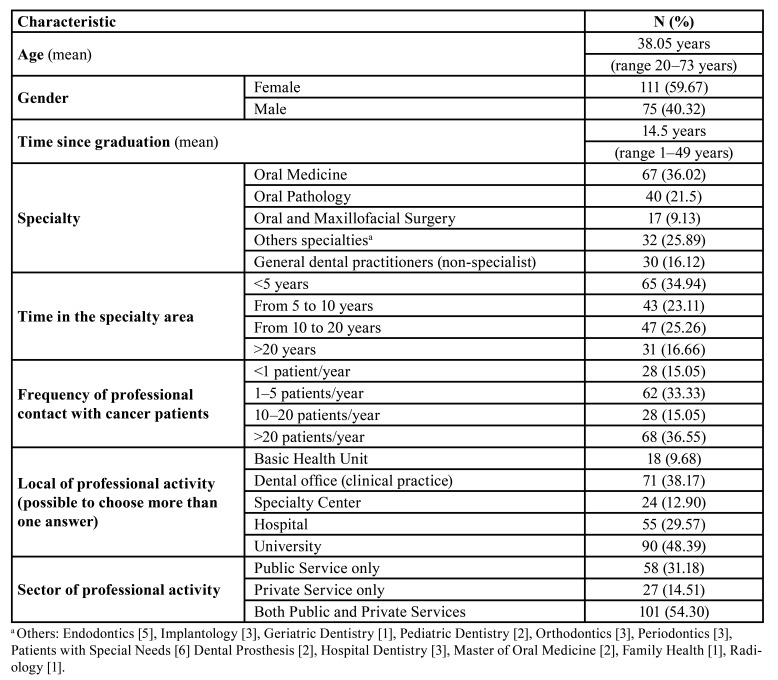
Demographic profile of dentists included in this study.

The mean number of years since graduation was 14.5 years (range 1-49 years). Regarding the number of years as a specialist, 34.94% worked in their specialty for 5 or more years. The time of practice in the specialty had a weak positive correlation with the level of confidence and satisfaction in the DBN (rho=0.226; *p*=0.002). We observed the same positive correlation regarding the time since graduation (rho=0.259; *p*<0.001).

The most frequent professional contact with cancer patients was >20 patients/year, or 68/186 (36.55%). Most respondents worked in universities (90/186 or 48.39%) and private practice (71/186 or 38.17%). Others reported a variety of workplaces. The Kruskal-Wallis test revealed the influence of the workplace on contact with cancer patients (H[2]=9.62; p=0.008) and confidence level to DBN (H[2]=16.65; p<0.001). The pairwise comparisons pointed out that participants working only in the private sector had less frequent contact with cancer patients than those working in the public sector (W=-4.03; p=0.012), and dentists working only in the private sector felt less confident about DBNs than those working in both sectors (W=−5.55; p<0.001).

The satisfaction levels with communication skills differed between professional specialties (H[3]=17.85; *p*<0.001). Pairwise comparisons showed that general dental practitioners (nonspecialists) reported less satisfaction with their communication skills than oral medicinists (W=−4.75; *p*=0.004) and oral pathologists (W=−5.15; *p*=0.002). The frequency of contact with oncological patients showed a significant difference (H[3]=28.9; *p*=0.001). Oral medicine doctors had more contact with cancer patients than nonspecialists (W=6.72; *p*<0.001). Additionally, oral medicine and oral pathology specialists more often reported having a consistent plan/strategy for DBN than general dental practitioners (X²[6]=23.3; *p*<0.001).

Table 2 shows the communication of cancer diagnosis in clinical routine. A total of 102 (52.84%) dentists reported communicating bad news to a cancer patient less than once/month, followed by 68 (36.56%) who gave a cancer diagnosis 1 to 5 times/month. When asked about receiving any specific training to communicate bad news, 82 (44.09%) answered that they observed professionals delivering cancer diagnoses, 25 (13.44%) had formal education (during university, training, or continuing education courses), 35 (18.82%) had both, and 44 (23.66%) had no specific training.

**Table 2:**
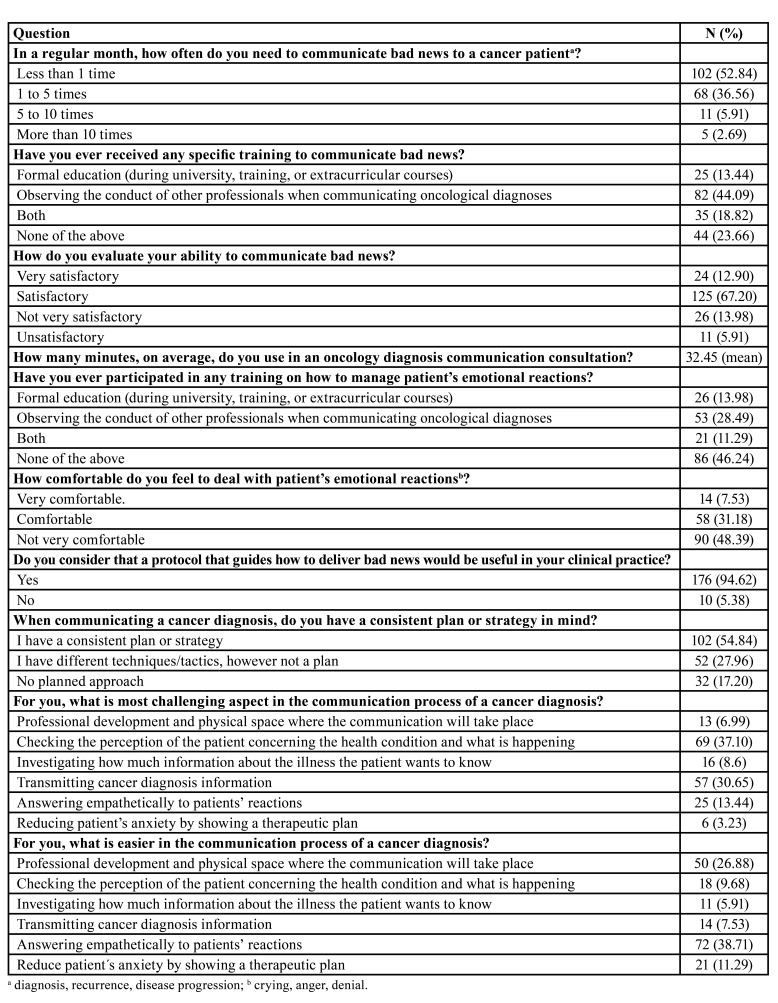
The communication context on cancer diagnosis in clinical routine.

The mean time used for a consultation to deliver a cancer diagnosis was 32.45 minutes. Regarding how comforTable dentists felt when dealing with patients’ emotional reactions, 90/186 (48.39%) reported discomfort. When asked if they had a clinical practice guide or a protocol to assist in the delivery of bad news, 176/186 (94.62%) respondents liked the idea. This survey showed that 102/186 (54.84%) dentists had a consistent plan or strategy to communicate a cancer diagnosis. Understanding the patient’s perception of the diagnosis was the most challenging task for 69/186 (37.10%) of respondents, and demonstrating empathy was the easiest (72/186 or 38.71%) part of the communication process of a cancer diagnosis.

**Figure 1:**
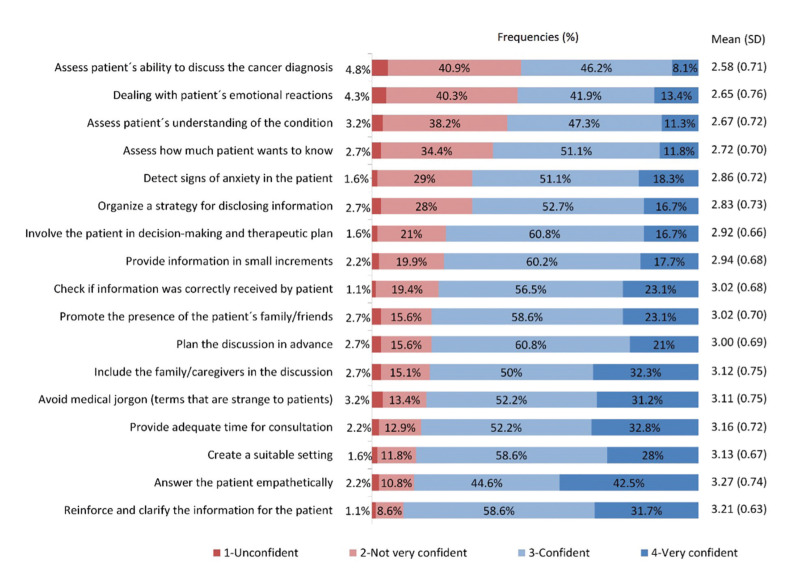
How confident dentists feel when performing each of the steps during a cancer diagnosis communication.

Fig. 1 reports the distribution of responses showing how confident the participant felt when performing each step during a cancer diagnosis communication. A total of 113 (60.75%) dentists felt confident in planning the discussion in advance, 109 (58.60%) created a suiTable environment for DBN, and 93 (50%) agreed with the presence of the patient’s family/friends. Of the total, 52 (27.96%) participants did not feel very confident in organizing a strategy to inform the patient about his/her condition, and 76 (40.86%) did not feel confident in assessing the patient’s ability to discuss the cancer diagnosis.

Most respondents, 112 (60.22%), felt confident in providing information to the patient gradually; 97 (52.15%) avoided medical jargon, and 109 (58.60%) reinforced and clarified the information for the patient. Regarding dealing with patients’ emotional reactions, 78 (41.94%) felt confident, and 83 (44.6%) did not feel confident or very confident. Most dentists were confident in involving the patient in the decision-making and therapeutic plan; 113 (60.75%) and 97 (52.15%) provided adequate time for consultation.

Statistical analysis with the Spearman coefficient showed a positive correlation between the frequency of professional contact with cancer patients and the confidence level in communicating the cancer diagnosis (rho=0.336; p<0.001), planning the discussion in advance (rho=0.440; *p*<0.001), assessing the patient’s understanding about his/her condition (rho=0.320; p<0.001), and organizing a strategy to deliver the information (rho=0.355; p<0.001). Respondents who reported not having a planned approach more often had no training experience, whereas professionals who followed the approach of other colleagues and those who received a formal education more often reported having a plan/consistent communication strategy (X²[6]=23.4; *p*<0.001).

## Discussion

Our study assessed, for the first time, the barriers faced by Brazilian dentists when communicating oral and oropharyngeal cancer diagnoses to patients. The study used a questionnaire based on the SPIKES protocol (10).

When communicating a cancer diagnosis, the information provided to patients should be based on the assessment of their clinical condition and on the understanding of the effects of sociocultural issues. Thus, the current literature has introduced a discussion of “Patient-Centered Care”, where specific health needs and desired health outcomes are the driving force behind all decision-making processes regarding the course of a disease (10). Recent evidence has shown that effective communication is associated with more favorable health outcomes (3). Empathic communication and active listening by professionals have been positively associated with patient satisfaction, less emotional distress, and greater perceived ability to deal with the disease and treatment (12).

In the course of the disease, the first signs and symptoms in patients with oral cancer are usually noticed by general dental practitioners (13). However, these professionals are reluctant to approach the topic (11,14). In our study, we observed that the greatest barriers faced by dentists were related to the possible reactions and emotions of patients when receiving bad news. Many dentists feel unprepared to discuss cancer with patients since they report difficulty dealing with this type of communication. Issues include lack of confidence to answer certain questions, lack of time during consultations, and fear of making patients anxious (15). In this context, our study found that 44.62% of respondents did not feel very confident or unconfident in dealing with patients’ emotional reactions. Moreover, 41.4% of the participants did not feel confident in assessing the patient’s understanding of the condition. Our study found that the topics covered in the questionnaire that are under the control of the professional, for instance, planning/strategy for communication and creating a suiTable setting, are the ones that dentists feel in general more confident. Although most dentists in our study feel very confident or confident with the topics discussed above, the number of participants who do not feel confident is still very high.

The minority of professionals who receive specific training during formation is a possible explanation. Overall, their reference is limited to their own clinical experience or observing the conduct of other professionals when communicating oncological diagnoses (15,16). Our data converge with the studies. Our study found that only 13.44% of the dentists had formal education (during university, training, or continuing education courses) in communicating bad news. Within dentistry, bad news communication goes beyond oral cavity/oropharyngeal cancer communication. For instance, patients in the clinic are often faced with dental decisions that can be life-changing. Challenges such as the loss of anterior teeth, fracture of teeth, and extractions can be devastating for the patient. These situations can also generate emotional reactions and affect the patient’s expectations and attitude toward the clinician, reinforcing the need to train dentists for challenging communication (17).

Therefore, knowing how to communicate bad news is essential for professionals; however, this knowledge should start with undergraduate students since they already face challenging situations in the clinic (17). Brazilian national curriculum guidelines for the undergraduate course in dentistry, in force since 2002, mentioned communication professional skill in simplistic terms, focused on confidentiality, interaction with other health professionals, the general public, and knowledge accuracy in a foreign language. A new resolution broadened this concept only in June 2021, including interaction with users and family members. Aspects of importance include empathy, sensitivity, interest, and respect for knowledge and popular culture by using accessible language, enabling users to understand the actions and procedures. Despite the lack of specific information concerning DBNs, expanding this concept in dentistry training in Brazil is a tendency (17). To improve the communication of dentists with patients in Brazil, teaching programs for undergrads (17) and trainings should be implemented (16).

Note that in our study, professionals who had no training experience more often reported not having a planned approach, while professionals who followed the approach of other colleagues and those who received a formal education more often reported having a communication strategy (*p*<0.001). Additionally, most of the respondents who participated in some type of training on how to manage patients’ emotional reactions were oral medicine doctors or oral pathologists (86 participants).

Our study demonstrated that general dental practitioners (nonspecialists) report less satisfaction with their communication skills than specialists. Oral medicine doctors and oral pathologists showed greater satisfaction with their communication skills when compared with other specialists (*p*<0.001). Oral medicine doctors were also more confident in their communication skills while DBN. The possible reasons for these findings could be that satisfaction and confidence are closely linked with knowledge, training process and experience that exposes the specialist to cancer patients during the process of communicating the malignancy diagnosis to the patient. We, like other authors, believe that all dental professionals should receive training to manage cancer patients (18-24).

In our study, 94.62% of the respondents considered that a protocol on how to deliver bad news would be useful in their clinical practice. Awojobi O. *et al*., 2016 evaluated the effect of a brief, focused training session on the use of an oral cancer communication guide for dentists (16). After training, dentists reported having more confidence discussing oral cancer with their patients, indicating that the training had a positive impact in reducing perceived barriers to oral cancer-related discussions, increasing self-efficacy, and increasing oral cancer discussions between dentists and patients (19).

Health professionals face issues when having to DBN. Studies have shown that professionals working with oncology have a high risk of experiencing burnout (25,26). They may encounter multiple stressors that could negatively influence their quality of life across time (27). Therefore, patients and health professionals will benefit if professionals are better trained in communication skills (28). Additionally, developing good communication skills with patients can reflect positively on the mental health of professionals (7).

In conclusion, our study found that dentists´ greatest barriers to DBN are associated with lack of confidence in dealing with patients´ emotional reactions and lack of training on this topic. Moreover, most dentists who participated in the survey believe that a protocol to guide the communication of bad news would be useful for clinical practice. For those protocols to be used by dentists, specific training is an important tool for these protocols to be incorporated by professionals, thus improving the confidence and satisfaction of these dentists for communicating bad news.
